# Focal dystonia and the Sensory-Motor Integrative Loop for Enacting (SMILE)

**DOI:** 10.3389/fnhum.2014.00458

**Published:** 2014-06-20

**Authors:** David Perruchoud, Micah M. Murray, Jeremie Lefebvre, Silvio Ionta

**Affiliations:** ^1^Laboratory for Investigative Neurophysiology, Department of Radiology and Department of Clinical Neurosciences, University Hospital Center and University of LausanneLausanne, Switzerland; ^2^The Electroencephalography Brain Mapping Core, Center for Biomedical ImagingLausanne, Switzerland

**Keywords:** sensory motor integration, modeling, TMS, fMRI, inhibition, neural plasticity, movement disorders, rehabilitation

## Abstract

Performing accurate movements requires preparation, execution, and monitoring mechanisms. The first two are coded by the motor system, the latter by the sensory system. To provide an adaptive neural basis to overt behaviors, motor and sensory information has to be properly integrated in a reciprocal feedback loop. Abnormalities in this sensory-motor loop are involved in movement disorders such as focal dystonia, a hyperkinetic alteration affecting only a specific body part and characterized by sensory and motor deficits in the absence of basic motor impairments. Despite the fundamental impact of sensory-motor integration mechanisms on daily life, the general principles of healthy and pathological anatomic–functional organization of sensory-motor integration remain to be clarified. Based on the available data from experimental psychology, neurophysiology, and neuroimaging, we propose a bio-computational model of sensory-motor integration: the Sensory-Motor Integrative Loop for Enacting (SMILE). Aiming at direct therapeutic implementations and with the final target of implementing novel intervention protocols for motor rehabilitation, our main goal is to provide the information necessary for further validating the SMILE model. By translating neuroscientific hypotheses into empirical investigations and clinically relevant questions, the prediction based on the SMILE model can be further extended to other pathological conditions characterized by impaired sensory-motor integration.

## INTRODUCTION

The ability to correctly perform movements in everyday life is critical to adequately interact with the environment. Several movement disorders manifest as remarkable deficiencies in the ability to control voluntary actions and interact successfully with the surroundings. The onset of the disease may, in fact, disrupt normal functioning of fundamental neural and cognitive processes that permit efficient sampling of sensory events from the environment in order to implement adequate motor routines. Sensory-motor integration refers to this ability to coherently organize bodily sensations and motor responses. Despite its crucial role as a proficient interface with the environment, current models of sensory-motor integration lack a definitive overview on the reciprocal interplay between sensory input and motor output. The opportunity to distinguish and properly understand the relative weight of sensory and motor processes is provided by mental imagery, a cognitive task that activates sensory-motor representations without physical sensory stimulation. This experimental approach is particularly important for studying the physiology of pathological conditions characterized by sensory-motor integration deficits but without impairments in basic motor functions, such as focal dystonia.

Dystonia is a disabling movement disorder characterized by involuntary muscle contractions frequently associated with abnormal movements and/or postures (Fahn et al., 1998). It is triggered or exaggerated by different voluntary movements, and often spreads over neighboring or corresponding muscles (Albanese et al., 2013). It is the third most common movement disorder after essential tremor and Parkinson’s disease (Breakefield et al., 2008). As a function of the distribution of the symptoms, dystonias can be classified as generalized (affecting almost the whole body) or local (affecting only some specific body regions, e.g., focal dystonia). Very frequent focal dystonias include spasms and abnormal postures of the eyelids (blepharospasm), articulatory apparatus (oromandibular dystonia or laryngeal dystonia), neck (cervical dystonia), or the hand (e.g., writer’s cramp). Focal hand dystonia (FHD) is one of the most common forms of focal primary dystonic disorders (Tarsy and Simon, 2006; Jankovic, 2009). It can be very heterogeneous and can affect only very specific tasks by inducing involuntary contractions – resulting in involuntary finger twisting or stretching – only in particular conditions (e.g., only while writing, typing, or playing musical instruments), but not in other manual activities. Despite steadily developing clinical procedures, little is known about its etiopathogenesis and our understanding of its pathophysiology is still insufficient (Zoons et al., 2011). For these reasons the treatment is often limited to only symptomatic therapy, such as focal application of botulinum toxin (Hallett et al., 2009). Botulinum toxin offers effective transient relief of symptoms. However, it requires elaborate injection schemes, is extremely time-consuming, and its efficacy drastically depends on the therapists’ experience. In addition, only 50% of FHD patients remain on treatment (Kruisdijk et al., 2007), and its long-term efficacy remains controversial (Dashtipour and Pender, 2008). Alternative therapeutic interventions such as non-invasive sensory-motor retraining can produce sustained improvements of motor functions in FHD (Byl et al., 2009). In particular, “sensory training” (Zeuner et al., 2002) and “sensory-motor retuning” (Candia et al., 1999) are therapeutic strategies focused on improving sensory-motor integration mechanisms. However, the underlying pathophysiological mechanisms involved in treatment-related behavioral and cortical changes remain to be clarified. Thus, before implementing new intervention protocols it is necessary to validate a model of sensory-motor integration, to investigate the pathology-dependent cortical sensory-motor organization, and to unravel the neurophysiological processes leading to treatment-induced cortical changes.

Despite the fact that different types of dystonia have long been considered as the result of impaired motor control, the focus has progressively been switched to the integration between sensory input and motor output. Basic research in neuroscience has produced converging evidence on the tight relationship between movement generation and recalibration (see also Todorov, 2004). Clinical research has also individuated the key role of sensory input (so-called alleviating maneuvers or sensory tricks) on ameliorating motor behaviors in several movement disorders (see also Abbruzzese and Berardelli, 2003). In the following sections we will describe the reciprocal role of deficits in sensory processing and abnormal motor control in different kinds of dystonia, starting from the description of the most significant abnormalities both at the behavioral and the neural level. Based on the reviewed data we will then propose and fully describe a comprehensive model of sensory-motor integration hereafter termed “Sensory-Motor Integrative Loop for Enacting (SMILE).” Accommodating the main three pathophysiological mechanisms of FHD [loss of inhibition (Hallett, 2011), aberrant neural plasticity (Quartarone and Pisani, 2011), and defective learning-based sensory-motor integration (Byl, 2007)], the SMILE model proposes plausible origin and feature of FHD and can be extended to other sensory-motor impairments.

## SENSORY-MOTOR DEFICITS IN FOCAL DYSTONIA

### BEHAVIORAL DATA

Behavioral data can be used to create general models, based on which it is possible to generate procedural hypotheses on the modules possibly involved in specific processes and their putative reciprocal connections in theoretical frameworks. Testing these hypotheses can contribute to individuate the origin of specific impairments and to assess early markers of a given disorder (Scontrini et al., 2009). In the case of dystonia, two main behavioral tasks have been largely used to conceive its pathophysiological mechanisms: the spatial discrimination threshold (SDT) and the temporal discrimination threshold (TDT).

#### Spatial characteristics

The SDT is a tactile task used to establish the minimal distance between two stimuli that participants can reliably discern as distinct events. Healthy subjects can detect changes in the orientation of tiny parallel grooves as thin as 1 mm when presented on the tip of the finger (Craig and Kisner, 1998). As an extension of the classical two-point discrimination task, the SDT can be assessed via the so-called “Johnson–Van Boven–Philips” domes. In this case, participants have to identify the orientation of a linear grating pressed on the skin. Starting from the hypothesis that dystonias are associated with an aberrant organization of the sensory cortex, Bara-Jimenez et al. (2000a, 2000b) used this technique among others, to compare the abilities of blindfolded FHD patients and healthy controls in localizing tactile stimuli delivered either to a single phalanx or to each individual phalanx of the right (dystonic) hand. The authors observed an impaired ability in FHD patients to discriminate grating orientation and demonstrated that spatial sensitivity was impaired in dystonic patients only when stimuli were delivered to different regions on the same phalanx. Sanger et al. (2001) replicated these results and additionally showed that SDT was also impaired in the non-dominant, non-symptomatic hand. To explore whether the impairments in SDT tasks are specific for FHD or are also detectable also in other types of dystonias, Molloy et al. (2003) conducted an experiment with domes applied bilaterally on the tip of the index fingers of patients suffering from either generalized or focal dystonia. Their findings contrast with the unspecific SDT impairment reported by Sanger et al. (2001). Generalized dystonia patients displayed similar performance compared to healthy subjects, while all focal dystonia patients showed impaired SDT. Importantly, only FHD patients showed a significant threshold difference between dominant and non-dominant hand.

In summary, the SDT has been largely used in populations suffering from a variety of dystonic disorders showing that in these conditions there are clear impairments not only in the motor components but also in sensory processing of spatial information coming from the affected body region. Beyond this first conclusion, on the basis of the available data it could be suggested that the disorganization due to FHD is not limited to a single body part but rather extends at least to the contralateral hand. However, it is still difficult to conclude whether the spatial discrimination impairments are restricted to only the symptomatic limb or are instead bilateral.

#### Temporal characteristics

Another frequently used task in dystonia-related research is the TDT, which identifies the minimal time interval between two stimuli that allows differentiating them as separate events. It typically involves unimodal electrical stimulation of the skin, but can be coupled, paralleled, or even replaced by visual, kinematic, or any other type of stimuli. On average, healthy subjects can discriminate two electrical stimuli on the index finger provided they are separated by at least 30 ms (Lacruz et al., 1991). In dystonic patients, a single or pair of non-noxious tactile stimuli applied to both index fingers elicit increased TDT (Tinazzi et al., 1999), further augmenting as a function of the distance between the stimulation sites (Tinazzi et al., 2002). Taking into account the potential effects of sensory modality and multisensory integration processing, Aglioti et al. (2003) extended the paradigm to visual–tactile stimulation in an investigation restricted to generalized dystonia patients. Using either electrical tactile stimulation of the index finger and/or visual stimuli with LEDs, they revealed increased TDT compared to healthy controls in all conditions, though particularly marked in the cross-modal situation. Additionally, they showed that temporal order judgments (i.e., the explicit reporting of the temporal order of several asynchronous stimuli) are also impaired in generalized dystonia patients (Aglioti et al., 2003). When conducting a similar experiment in FHD patients, the TDT for unimodal visual stimuli resulted in similar performance between patients and controls (Fiorio et al., 2003). Similar results have been obtained in cervical dystonia (Tinazzi et al., 2004) and blepharospasm (Fiorio et al., 2008) in contrast to corresponding non-dystonic patients (i.e., cervical pain and hemifacial spasms, respectively), suggesting that the impairment is selective for dystonic disorders. In order to check the specificity on increased TDT, Scontrini et al. (2009) stimulated either the hand, neck, or eyebrow in 82 focal dystonia patients including blepharospasm, FHD, cervical, and laryngeal dystonia. They observed a general increase of the discrimination threshold for all the investigated body parts. This corresponds with a study in which abnormalities in TDT during uni- and multi-modal visual–tactile processing were shown to be linked to the non-fully penetrant gene in both manifesting and non-manifesting carriers (Fiorio et al., 2007a).

In summary, dystonic patients show evidence of abnormalities not only in spatial discrimination, but also in temporal processing. Temporal discrimination seems to be affected both at symptomatic and non-symptomatic body regions. The reviewed data suggest a critical difference in the mechanisms of FHD and generalized dystonia. FHD patients’ impairment appears to be linked to tactile processing and visual–tactile integration, whereas the generalized dystonia patients exhibit more general impairments in integration processing, including exclusively visual processing of stimuli near the hands. Overall, most studies present a coherent picture of the relationship between dystonia and TDTs, whose increase in focal dystonia is specifically selective for sensory processing but not isolated to the symptomatic limb.

#### Kinesthetic impairments in dystonia

It has been demonstrated that the movement induced by tonic vibration of a tendon is impaired in generalized dystonia (Tempel and Perlmutter, 1990). Grünewald et al. (1997) assessed the properties of this effect in focal dystonia. Participants were blindfolded and asked to mimic the movements of one arm with the other arm. The “master” arm was either moved passively by the experimenter, or movement was induced by means of tonic vibrations at the level of the biceps tendon. Focal dystonia patients could accurately track passive movements. However, and unlike healthy subjects, tracking during induced movements was impaired, even if the vibration-induced flexion was normal (Grünewald et al., 1997). With respect to other movement disorders, this impairment is specific for focal dystonias (Rome and Grunewald, 1999), regardless of the stimulated body segment (Yoneda et al., 2000). The detection of postural changes is preserved in the passive condition but not in the induced condition. These results suggest a deficit in processing the sensory feedback of a muscular contraction (as in the induced condition), while the perception of position per se (proprioception, as in the passive condition) would remain intact (see also Frima et al., 2003, 2008). This interpretation is in line with recent clinical evidence showing the key role of altered proprioceptive feedback in FHD (Konczak and Abbruzzese, 2013).

### STRUCTURAL IMAGING

Few studies investigated the structural brain organization of FHD and the available data are largely inconsistent. Some studies associated FHD with anatomical abnormalities at the cortical level (Garraux et al., 2004; Delmaire et al., 2007), some others to sub-cortical irregularities (Draganski and Bhatia, 2010; Granert et al., 2011b). In particular, part of the evidence from structural brain imaging on the pathophysiology of dystonia highlights the role of aberrations in subcortical structures, including the basal ganglia (Bhatia and Marsden, 1994; Krystkowiak et al., 1998; Draganski et al., 2009; Beukers et al., 2011), mesencephalon (Vidailhet et al., 1999), and the cerebellum–thalamus–cortex axis (Argyelan et al., 2009). Conversely, other studies associated FHD with structural anomalies in the sensory-motor (Garraux et al., 2004; Delmaire et al., 2007) and the premotor cortex (Granert et al., 2011a). Even the directionality of volumetric differences between FHD patients and controls does not provide a straightforward method for individuating a precise neural substrate responsible for – or at least associated with – the symptoms. With respect to healthy controls, in FHD patients the gray matter volume of a wide range of regions has been considered either increased or decreased. This network includes the putamen [increased (Black et al., 1998; Bradley et al., 2009), decreased (Obermann et al., 2007)], thalamus [increased (Obermann et al., 2007), decreased (Delmaire et al., 2007)], cerebellum [increased (Draganski et al., 2003), decreased (Delmaire et al., 2007)], prefrontal cortex [increased (Egger et al., 2007), decreased (Draganski et al., 2003)], and inferior parietal cortex [increased (Etgen et al., 2006), decreased (Egger et al., 2007); **Table 1**].

**Table 1:** Dystonia-related morphometric changes.

Region	Increased volume	Decreased volume
Prefrontal cortex	Egger et al. (2007)	Draganski et al. (2003)
Inferior parietal lobe	Etgen et al. (2006)	Egger et al. (2007)
Cerebellum	Draganski et al. (2003)	Delmaire et al. (2007)
Thalamus	Obermann et al. (2007)	Delmaire et al. (2007)
Putamen	Bradley et al. (2009)	Obermann et al. (2007)

*Previous studies have reported conflicting results in volumetric properties of cortical areas, basal ganglia, and cerebellar regions.*

In general, one of the main reasons of such inconsistencies might be the use of different scanners, data recording sequences, and analysis procedures. One possibility for overcoming this heterogeneity may be the use of quantitative methods that are more conducive to comparisons across laboratories/scanners, such as Voxel-Based Quantification (Draganski et al., 2011). This automated unbiased analysis technique overcomes previous limitations including whole-brain multi-parameter mapping at high resolution, correction of radio-frequency inhomogeneities (Lutti et al., 2010), and diffeomorphic registration (Ashburner, 2007). Previous work demonstrated parameter-specific distribution patterns in healthy ageing and suggested a biophysical interpretation in line with histological studies demonstrating age-dependent iron accumulation and pathological rate of de-/re-myelination (Bartzokis et al., 2007a, b). Taking advantage of the use of voxel-based quantification, future studies will be able to precisely identify the anatomical neural correlates of FHD and other types of dystonia.

### FUNCTIONAL IMAGING

Using passive vibrotactile stimulation of single fingers, functional neuroimaging studies have described the disorganization of the somatosensory representations in dystonia. Nelson et al. (2009) found that in FHD patients the representations in primary sensory cortex (S1) relative to the fingers involved in writing are overlapping and spatially less separated, while no difference is observed for the other fingers with respect to healthy controls. In addition to the decreased separation of the finger representations in S1, Butterworth et al. (2003) reported that in FHD the order of the representations in S2 is inverted and activations are weaker with respect to healthy controls. However, taking into consideration the extreme task-specificity of several kinds of dystonias, it would be important to consider the fine-tuned loop between specific movements and precise sensory feedback, instead of pointing to the sensory deficits as the only origin of dystonic disorders. Following this line, several neuroimaging studies investigated the neural correlates of active movements in FHD by asking patients to physically perform a movement while functional magnetic resonance imaging (fMRI) data were recorded. In order to test the hypothesis that a dysfunctional balance between neighboring finger representations could be one origin of FHD, a recent study required FHD patients to control a cursor on a screen by regulating the force of a single movement involving only one finger and a coupled movement involving two fingers of the affected hand (Moore et al., 2012). In FHD patients the coupled movements were associated with decreased activity in bilateral S1, right parietal cortex and cerebellum, and left putamen. Conversely, no differences were observed for the single movement with respect to healthy controls. Based on this data, it can be concluded that in FHD only the coupled movements are specifically affected and it might be further hypothesized that the pattern of cerebral activity would vary as a function of movement difficulty. Accordingly, FHD patients have been asked to use the affected hand to either write (complex movement) or flex/extend the fingers (simple movement) while fMRI data were recorded (Havrankova et al., 2012). Consistent with Moore et al. (2012), Havrankova et al. showed the hypoactivation of S1 and parietal cortex. However, no involvement of cerebellum or basal ganglia was reported. Additional investigations on the potential influence of movement complexity showed premotor hyperactivity and cerebellar hypoactivity associated with unimanual and bimanual finger tapping in FHD patients (Kadota et al., 2010). Hu et al. (2006) asked FHD patients to perform progressively more complex kinds of writing while being in the fMRI scanner and, with respect to healthy controls, they found increased activation in motor cortex, basal ganglia and cerebellum associated with complex writing (using the pen) but no differences for simple writing (using the finger). This would support that movement complexity plays a central role in the symptoms exhibition and the relative cerebral activity.

Despite an initial general agreement, the level of inconsistency in terms of affected key regions between different studies increases as slightly different tasks are taken into account. Indeed if FHD patients are asked to *physically perform* movements, neural activity has been reported to be abnormal in a very heterogeneous network, including basal ganglia (Chase et al., 1988; Siebner et al., 2003; Blood et al., 2004; Peller et al., 2006; Schneider et al., 2010), thalamus (Preibisch et al., 2001; Hu et al., 2006), sensory-motor cortex (Preibisch et al., 2001; Islam et al., 2009; Jankowski et al., 2013), supplementary motor area (Oga et al., 2002; Hu et al., 2006), prefrontal cortex (Playford et al., 1998; Pujol et al., 2000; Preibisch et al., 2001; Dresel et al., 2006), and primary motor cortex (M1; Ceballos-Baumann et al., 1995; Playford et al., 1998; Ibanez et al., 1999; Pujol et al., 2000; Detante et al., 2004; Dresel et al., 2006). However, there is strong evidence supporting the position that the sensory feedback during movement execution is altered in FHD and other forms of focal dystonia (see Defective Learning-based Sensory-Motor Integration). For example, by asking patients to perform symptomatic and asymptomatic movements while fMRI data were recorded, Simonyan and Ludlow (2010) found abnormal activity not only in the brain regions encoding the motor command but also in the network processing the sensory feedback including S1, insula, basal ganglia, and thalamus. Based on these data, the functional neuroimaging studies that investigated the features of sensory-motor representations in focal dystonia by asking patients to physically perform a movement might be affected by the confound due to the altered sensory feedback associated with movement execution itself. One possible way to overcome this limitation is to investigate the pattern of neural activity at rest. The comparison of the correlation between activity changes in different brain areas during different tasks and rest brought to the scientific community one of the most robust findings throughout more than a decade of neuroimaging science: the implication of the medial prefrontal cortex, inferior parietal cortex, and precuneus in a canonical network dubbed as the “default mode network” (e.g., Raichle et al., 2001; Buckner et al., 2008; Gultepe and He, 2013). Investigating the properties of the default mode network in FHD patients, Mohammadi et al. (2012) found that with respect to healthy controls, FHD patients show reduced connectivity in postcentral regions and augmented connectivity in basal ganglia. These data speak in favor of a disorganization at the level of the sensory-motor system, in particular the basal ganglia and the somatosensory cortex; both important for coding the afferent sensory feedback. However, despite the undisputed advances brought by the resting-state approach in circumventing potential confounds due to altered sensory feedback, it still does not provide information on the origin of task-specificity, one of the most peculiar aspects of FHD (see Mental Imagery and Rotation as “Clean” Tools to Investigate Sensory-Motor Mechanisms).

## POTENTIAL MECHANISMS OF FHD

### LOSS OF INHIBITION

For several decades, the excitatory/inhibitory regulations of the central nervous system have been proposed as impaired in both generalized dystonia and FHD (Tinazzi et al., 2009). Atypical excitability and activity would result in the deterioration of the communication pathways between the central nervous system and the periphery. Nevertheless, testing this type of hypothesis is particularly challenging using conventional neuroimaging or behavioral techniques, due to the difficulty of distinguishing between excitatory or inhibitory processes. In order to overcome this limitation transcranial magnetic stimulation (TMS) – a non-invasive technique allowing the excitation or inhibition of specific brain regions through magnetic pulses – has been largely used to study the properties of given cortico-spinal pathways (Miniussi and Thut, 2010) both in healthy and clinical populations (Brodbeck et al., 2010; Rotenberg, 2010). The features of the “motor-evoked potentials” (time-locked electromyographic activity resulting from a supra-threshold TMS pulse over the motor cortex) and cortical silent period (CSP; the interval of silent electromyographic activity following a supra-threshold TMS pulse) can provide information regarding the underlying state of the neural populations. The duration of CSPs for TMS depends on the recording site, the intensity of the TMS with respect to the motor threshold, and the onset considered as the starting point (i.e., absolute versus relative CSP). In general, the typical CSP oscillates within a range from 110–140 ms (Orth and Rothwell, 2004) to 160–170 ms (Aurora et al., 1999; Priori et al., 1999). In FHD the (a)typical CSPs are shortened (Kimberley et al., 2009), restricted to the symptomatic hand (Chen et al., 1997), and task-specific (Tinazzi et al., 2005b). For example, Tinazzi et al. (2005a) used CSP together with a facilitation/rest electromyographic motor-evoked potentials to demonstrate the task-specific motor impairment of FHD. In this study, while TMS was delivered and motor-evoked potentials were recorded, participants performed both pincer grip (a finely tuned contraction of only the thumb and index finger) and power grip (a co-contraction of all fingers). With respect to healthy controls, FHD patients had different CSP and motor-evoked potentials depending on the type of grip performed. In particular, while pincer grip elicited shorter CSP and larger motor-evoked potentials amplitude ratio, power grip remained unchanged, supporting the specificity of excitatory/inhibitory impairment mechanisms in FHD (see also Kimberley et al., 2009).

In addition to CSP, other types of inhibition features are potent markers of neural pathway mechanisms, and have been shown to present abnormalities in all types of dystonia at the level of both the central and the peripheral nervous system (Hallett, 2006; Lin and Hallett, 2009). At the central level, intracortical surround inhibition (the capacity of an excited neuron to reduce the activity of the neighbors) is decreased in FHD (Ridding et al., 1995; Chen et al., 1997; Espay et al., 2006; Lin and Hallett, 2009). At the peripheral level, reciprocal inhibition (the coordinated contraction and relaxation of agonist and antagonist muscles, respectively) is dramatically impaired in FHD patients (Nakashima et al., 1989; Panizza et al., 1990).

Animal studies showed that aberrant intracortical surround inhibition can lead to dystonic behaviors (Matsumura et al., 1991, 1992). In humans such loss of inhibition can be investigated using TMS. For example, Sohn and Hallet (2004) set the TMS as targeting the portion of M1 corresponding to the little finger, but triggered by the activity elicited by self-initiated flexion of the index finger. Using this approach the authors investigated surround inhibition in FHD patients by evaluating the little finger reactivity during volitional flexion of the index finger. Their results showed that in FHD patients the motor-evoked potentials’ amplitude was increased.

In addition to intracortical surround inhibition, also interhemispheric inhibition (the ability of a unilateral hemispheric stimulation of the motor cortex to inhibit the contralateral motor cortex given a short latency) is impaired in FHD (Sohn and Hallet, 2004; Beck et al., 2009). Interhemispheric inhibition is usually investigated with dual-site TMS, where a conditioning stimulus is applied in one hemisphere, and shortly followed by a test stimulus in the corresponding sensory-motor area of the contralateral hemisphere. In healthy controls the conditioning stimulus has a suppression effect over the test stimulus (Perez and Cohen, 2009). Analyzing the amplitude of motor-evoked potentials of this test-pulse allows the investigation of the underlying modulation of interhemispheric inhibition. Beck et al. (2009) showed that interhemispheric inhibition is partially lost in patients with mirror dystonia (consisting of dystonic behaviors in one hand when acting with the other hand), while non-mirror dystonia patients exhibited similar performance compared with healthy subjects. This discovery suggests that interhemispheric inhibition is not deeply involved in the basic pathophysiology of dystonia, but only in its mirror aspect. In order to investigate the task-specificity of inter-hemispheric inhibition in mirror dystonia, Sattler et al. (2013) extended the previous study with a rest versus pen-holding task. At rest, the inter-hemispheric inhibition levels of all three groups (healthy subject, mirror and non-mirror FHD) were similar, but mirror patients displayed a large bilateral decrease in inter-hemispheric inhibition in the pen-holding condition, inversely related to the severity and duration of symptoms. Altogether, these two studies agree on the involvement of impaired inter-hemispheric inhibition only in mirror dystonia.

Some groups focused on psychogenic dystonia, a type of dystonic disorder without a clear neurological basis and possibly associated with other psychological disorders. In this vein, Espay et al. (2006) used TMS for investigating a broad range of behavioral features in both psychogenic and organic (non-psychogenic) dystonia. These features included reciprocal inhibition, CSPs, but also cutaneous silent period, as well as short- and long-intracortical inhibition. All of these behavioral markers were statistically different between healthy subjects and dystonia patients. The only statistically relevant difference between behavioral results in psychogenic and organic dystonia involved reciprocal inhibition.

Altogether these data suggest that different types of dystonia, whether primary or secondary to psychological disorders, share basic mechanisms as well as widespread cortical and subcortical abnormalities. However, the question whether these excitatory/inhibitory regulation abnormalities common to several types of dystonias are a cause or a consequence of the disorders still needs to be resolved. Nonetheless, some studies have linked dystonic symptoms to abnormal activity of numerous modules in the basal ganglia–thalamocortical circuit (Vitek, 2002; Liu et al., 2008). While synchronous neural activity is involved in the planning and execution of movement in healthy subjects, dysregulation in the degree of synchronization might disrupt the proper function of the sensory-motor feedback system as a whole (Schnitzler and Gross, 2005). In addition, despite the limited available data, some individual case reports suggest the involvement of lesions in the pallidal–thalamus complex (Krystkowiak et al., 1998; see also Quartarone and Hallett, 2013). Accordingly, a single-subject study indicated that in situ electrical stimulation (“deep brain stimulation”) of the thalamus can ameliorate dystonic behaviors in FHD (Fukaya et al., 2007). However, due to the limited amount of data, it is difficult to clearly define the role of the pallidal–thalamus complex.

### ABNORMAL NEURAL PLASTICITY

Neural plasticity includes, but is not limited to, the ability of the brain to re-organize its neural connections as a function of both internal and external factors. Animal studies showed that over-trained repetitive movements abnormally remodels somatosensory cortical maps, leading to sensory de-differentiation between the receptive fields of neighboring digits (Byl, 2007). This de-differentiation parallels the development of dystonic-like behaviors (Byl et al., 1996; Blake et al., 2002). In other words, after a prolonged and intense stimulation, the neuron which previously coded the sensory input relative to only one finger starts to respond to sensory inputs delivered to more fingers (Byl et al., 1997).

Indeed, dystonia-related changes of receptive field features have been reported in sub-cortical structures such as the globus pallidus and thalamus (Lenz et al., 1998), key nodes in the generation of sensory and/or motor representations. Such neuro-plastic changes would be at the basis of aberrant pairing of tactile stimuli in healthy subjects (Godde et al., 1996), and associated with excessively repeated movements in FHD patients (Roze et al., 2009; Altenmuller and Jabusch, 2010). Experimental evidence showed that FHD is associated with finger de-differentiation in S1 and S2 (Butterworth et al., 2003; Nelson et al., 2009), basal ganglia (Rothwell and Huang, 2003; Quartarone et al., 2008), and cerebellum (Thompson and Steinmetz, 2009). Interestingly, non-manifesting carriers of a gene supposed to be involved in developing dystonia exhibit impairments in sequence learning but not in motor learning in general (Ghilardi et al., 2003). This supports the proposition that dystonia is a complex disorder due to aberrant integration mechanisms, biologically based on abnormal neuronal plasticity as a predisposing endophenotypic trait (Quartarone and Pisani, 2011).

The so-called “paired-associative stimulation” approach can be used to induce and identify the characteristics of neural plasticity. During paired-associative stimulation a given sensory stimulus is paired with TMS of a specific brain region, creating an artificial and relatively long-term association between an external event and the TMS pulse (Rizzo et al., 2009). To measure the excitability of the target region, evoked potentials are often recorded before and after the pairing. The kind of evoked potentials can vary according to the experimental protocol and the stimulated brain area: auditory (Schecklmann et al., 2011), somatosensory (Litvak et al., 2007; Pellicciari et al., 2009) or motor (Huber et al., 2008) evoked potentials. For example, similarly to the sensitivity of long-term potentiation (timing-dependent changes of synaptic efficacy) to specific associative processes both in the human (Cooke and Bliss, 2006) and other mammals’ brain (Bliss and Lomo, 1973), Litvak et al. (2007) demonstrated that paired-associative stimulation performed with TMS of S1 delivered near-synchronously to median nerve stimulation induced changes in the somatosensory evoked potentials correlated with behavioral changes in tactile discrimination abilities. Using median nerve stimulation and TMS over S1 as paired-associative stimulation, Tamura et al. (2009) likewise showed that in FHD patients the waveform elicited by TMS increased immediately after paired-associative stimulation, suggesting an abnormally increased excitability of S1. Theta-burst stimulation – a repetitive TMS protocol in which short trains of high-frequency magnetic pulses are repetitively discharged to modulate the short-term excitability level of a given brain area (Cardenas-Morales et al., 2010) – can also be used to induce plastic changes. Based on the emerging hypothesis that a cerebellar dysfunction might be tightly linked to the development of focal dystonia (Raike et al., 2012), Hubsch et al. (2013) used theta-burst stimulation of the cerebellum to investigate how its excitability can influence neural plasticity of M1 induced by paired-associative stimulation in FHD patients. After performing intermittent (excitatory) or continuous (inhibitory) theta-burst stimulation of the cerebellum, the authors paired the stimulation of M1 and of the median nerve both at 5Hz for intervals of 2 minutes. Results showed a complete loss of cerebellar influence on M1 plasticity, specifically for FHD. In the same study, the authors also showed that FHD patients had both lower performance in learning a new task and in “washing out” a previously learned task in order to adapt to a modification. These data suggest that the loss of cerebellar influence on sensory-motor cortex might be linked to atypical neural plasticity.

### DEFECTIVE LEARNING-BASED SENSORY-MOTOR INTEGRATION

According to the defective sensory-motor learning hypothesis, the different types of dystonia would be characterized by functional alterations in the sensory-motor circuit supposed to integrate sensory input and motor output (Breakefield et al., 2008). In this view the dystonic behavior could be due to abnormal somatosensory feedback received by the motor system during the movement. In this vein, it has been shown that over-practice can cause an overlap of the somatosensory receptive fields (Butterworth et al., 2003), which could lead to altered sensory representations and therefore to abnormal motor behaviors. In favor of this hypothesis, there is evidence that somatosensory finger representations in FHD patients are spatially closer (Bara-Jimenez et al., 1998), providing the biological justification to the notion that FHD develops in conjunction with excessive sensory stimulation or over-repetition of motor tasks (Quartarone et al., 2006).

The aberrant sensory input would be due to the disorganization of S1 (Hinkley et al., 2009). The overlap of digit representations in S1 would lead to excessive gain in the sensory-motor loop, due to the incongruence between the somatosensory and motor maps (Sanger and Merzenich, 2000). This incongruence would lead to a saturation of motor commands resulting in the dystonic movement of the affected hand or even in the muscular over-contraction and consequent paralysis. In this way the altered sensory representations would lead to abnormal motor behavior, highlighting the importance of sensory-motor integration. The critical role of the sensory feedback in modulating motor responses (Abbruzzese and Berardelli, 2003) is demonstrated by evidence showing how sensory discrimination is impaired in patients suffering from writer’s cramp (Sanger et al., 2001) as well as by the altered sensory-motor integration mechanisms in patients presenting musician’s dystonia (Rosenkranz et al., 2000) and writer’s cramp (Murase et al., 2000). In addition to experimental data, the importance of sensory processing in a movement disorder such as FHD is also demonstrated by the effectiveness of sensory re-training procedures (Zeuner et al., 2002). Despite short-term duration and reversibility, FHD patients can significantly improve their spatial acuity by performing daily sessions of Braille reading sessions for 8 weeks (Zeuner and Hallett, 2003).

However, the nature of the relationship between disorganized somatosensory information and aberrant motor output is still under debate. One possible explanation is that long-lasting non-physiologic motor behavior can cause changes in somatosensory representations. Alternatively, abnormal somatosensory representations may lead to abnormal motor output explaining the particular dystonic phenotype. Consequently, one of the main focuses for future research will be to investigate movement mechanisms in FHD and other types of movement disorders, but ruling out any confounding effect due to abnormal sensory feedback.

## MENTAL IMAGERY AND ROTATION AS “CLEAN” TOOLS TO INVESTIGATE SENSORY-MOTOR MECHANISMS

### MENTAL MOTOR IMAGERY

To identify the origin of dystonic behaviors it is crucial to understand the features of sensory-motor integration mechanisms while avoiding any potential confound due to altered sensory feedback. One possibility to achieve this goal is to use an investigation tool that does not require movement execution. This would help differentiate the mechanisms related to altered sensory feedback from those related to abnormal sensory-motor representations. Mental imagery is a cognitive task with such characteristics. In healthy subjects physical execution and mental imagery of a movement – “motor imagery” – share similar temporal and kinematic properties (Sirigu et al., 1996). The association between the properties of executed and imagined movements is further demonstrated by clinical studies showing how physical impairments are reflected in mental imagery. For example, if patients suffering from hemi-Parkinson’s disease are asked to physically perform and mentally imagine specific manual movements with the affected and the unaffected hand, the response times of the imagery task will be proportional to the asymmetries in the physical task; that is longer latencies for the affected than the unaffected hand (Dominey et al., 1995). Some data described the effects of FHD on motor imagery of different movements. In particular, in order to understand whether the physical impairments due to FHD generally or specifically influence the characteristics of mental imagery, patients suffering from writer’s cramp were asked to physically perform and mentally imagine finger tapping and writing (Tumas and Sakamoto, 2009). Surprisingly, with respect to healthy controls, patients had longer motor imagery latencies for both actions, suggesting that FHD would lead to unspecific deficits in mental imagery of complex movements.

In healthy controls, physical movement and motor imagery engage partially overlapping brain networks (Grezes and Decety, 2001). In particular, physical practice modulates the imagery-related brain activity in a specific network including the supplementary motor area, basal ganglia, and cerebellum (Ionta et al., 2010a). Several data support that also in clinical populations there is an association between the performance in motor imagery and the quantity or quality of neural activity. For example if Parkinson’s disease patients are asked to physically perform and mentally imagine hand and wrist movements, they show longer latencies and decreased activation patterns in fronto-parietal regions (Samuel et al., 2001). In addition, if Parkinson’s patients with freezing of gait perform motor imagery of walking, with respect to healthy controls their response times are longer and brain activity is decreased in the supplementary motor area and increased in the mesencephalic locomotor region (Snijders et al., 2011).

Combined with the manipulation of cortico-spinal excitability by means of TMS, motor imagery can be used to investigate not only the properties of cortical representations but also the characteristics of the communication between the central nervous system and the periphery. In particular, in healthy subjects top-down imagery-related mechanisms regulate the excitability of the sensory-motor pathways (Fourkas et al., 2006). In Parkinson’s patients the typical cortico-spinal excitability in response to the imagination of a movement is drastically reduced with respect to healthy controls (Tremblay et al., 2008). With regard to FHD patients, Quartarone et al. (2005) delivered the TMS pulse while participants were imagining index flexion. Similarly to the results shown by Sohn and Hallet (2004) on movement execution, during motor imagery the amplitude of motor-evoked potentials of all recorded hand and forearm surround muscles was increased in FHD patients, even for the arm not involved in motor imagery (Quartarone et al., 2005). This highlights the broad and less focused muscular activity in FHD patients compared with healthy subjects, even in the case of simply imagined movements.

Despite the differences with respect to the typically hyperkinetic dystonic disorders, a final example of the validity of implementing motor imagery protocols to evaluate motor excitability in focal dystonia, can be individuated in a study performed by Liepert et al. (2011). In this study, single and double TMS pulses were delivered while patients suffering from flaccid leg paresis due to psychogenic dystonia imagined ankle flexion. The amplitude of motor-evoked potentials resulting from the TMS pulse over the foot/leg motor cortex decreased with respect to rest, while it increased in healthy subjects (Liepert et al., 2011). This finding suggests an amplification of motor-imagery-related cortical excitability. Interestingly, during ankle movement observation on a video, motor-evoked potentials modulation of both healthy controls and psychogenic dystonia patients were similar (Liepert et al., 2011), emphasizing the difference between self-referred motor mechanisms and other-oriented visually based processing.

Only few brain imaging studies investigated the neural circuits recruited by motor imagery and their task-dependent activity in FHD. Despite the clear difference between primary and secondary dystonias, and as an additional example of the implementation of motor imagery as an investigation tool in dystonia-related disorders, Lehéricy et al. (2004) asked post-stroke secondary FHD patients to execute and imagine simple wrist flexion/extension while fMRI data were recorded. This study showed abnormal activity in parietal and frontal regions in patients with respect to controls during both motor imagery and execution. Similarly, patients presenting FHD secondary to complex regional pain syndrome showed abnormalities in the activity of fronto-parietal cortex during motor imagery of wrist flexion/extension (Gieteling et al., 2008). However, since both these studies focused on secondary dystonia, the results will not be further discussed but they can still be taken as demonstrations of the methodological reliability of the combination between motor imagery and neuroimaging for studying the neural correlates of dystonic behaviors.

At present the only available data on the neural correlates of motor imagery in primary FHD have been recently reported in two paired studies. In these studies FHD patients were asked to perform motor imagery of grasping a pencil with the purpose of either writing or sharpening it (Delnooz et al., 2012, 2013). In the first study the authors individuated the pattern of local of brain activity analyzing the modulations of the hemodynamic response, and showing that with respect to controls, FHD patients had stronger activity in premotor areas during imagery of grasping for writing but not during imagery of grasping for sharpening (Delnooz et al., 2013). These data suggest that in a region typically involved in balancing the motor output as a function of the sensory feedback, some degrees of abnormalities already exist at the level of movement planning or calibration. In the second study the authors applied a functional connectivity approach to the same dataset to further understand the interplay between the previously individuated regions of interest (Delnooz et al., 2012). This analysis showed that FHD patients had reduced connectivity between the premotor cortex and the parietal cortex with respect to controls (Delnooz et al., 2012). Taking into account that in healthy controls the coupling between premotor and parietal cortices is important for movement simulation and calibration (de Lange et al., 2006) and that the parietal cortex is an important hub for integrating information coming from different modalities (e.g., visual and motor; Fogassi and Luppino, 2005), the reduced functional connectivity between parietal and premotor cortex could be associated with a decreased ability to sample sensory feedback and integrate it with movement execution. However, these results should be considered with caution in the absence of a quantitative measurement of the patients’ imagery.

### MENTAL ROTATION

A straightforward way to control for and quantitatively measure motor imagery is provided by mental rotation, according to which people mentally rotate visually presented stimuli while response times and accuracy are measured. In healthy subjects the response times required to mentally align a stimulus to the vertical are a function of stimulus orientation (Shepard and Metzler, 1971; Parsons, 1994; Coslett et al., 2010). Mental rotation of body parts is specifically sensitive to bottom-up proprioceptive information (Ionta et al., 2007; Ionta and Blanke, 2009) and stimulus-driven strategies (Ionta et al., 2012) as well as to top-down cognitive regulations (Ionta et al., 2010b). These contributions highlight the twofold nature of sensory-motor processing (bottom-up and top-down influences) and the reliability of mental rotation as an investigation tool.

Based on this evidence, at least two motivations support the implementation of mental rotation in effective experimental protocols. First, as a motor imagery task, mental rotation supplies access to sensory-motor representations without any confounding effect potentially due to sensory feedback during movement execution. Second, providing quantitative measurements of the subjects’ performance, it adds important information on an otherwise purely introspective process. Thanks to these characteristics it has been used in several clinical populations. In particular, when asked to mentally rotate hands, patients suffering from cerebral palsy show the typical modulation of response times as a function of the stimulus orientation, but doubling the latencies with respect to healthy controls (Craje et al., 2010). This suggests that the bodily properties are spatially preserved but temporally impaired, probably because of the lack of use.

In addition, patients who lost their dominant limb due to amputation, show longer latencies and lower accuracy in the mental rotation of images depicting the amputated hand (Nico et al., 2004), therefore presenting highly specific impairments. The debate on the specificity of the effects has been further addressed taking into account the mental rotation performance of patients in which one or both hands never developed from birth, i.e., bilateral or unilateral amelia (Funk and Brugger, 2008). As in cerebral palsy, bilateral amelia results in a general slowing down, but does not affect the general modulation of the response times as a function of the stimulus orientation. Similarly to amputees, unilateral amelic patients’ performance is slower for the missing hand with respect to the present hand.

In cervical dystonia – affecting the neck muscles and then altering the vestibular input (Dauer et al., 1998; Karnath et al., 2000) – mental rotation of any body part is impaired (Fiorio et al., 2007b). Conversely, in FHD – affecting only one specific body region – the mental rotation of only the affected hand is selectively impaired (Fiorio et al., 2006). In a later study Katschnig et al. (2010) used mental rotation to investigate the differences between mobile and fixed dystonia. While mobile dystonia is typically characterized by involuntary task-specific muscle contractions (Berardelli et al., 1998), fixed dystonia results in immobile and persistent postures that do not regress even at rest (Schrag et al., 2004). Showing that patients presenting mobile dystonia (less debilitative) obtain shorter latencies with respect to fixed dystonia patients (more debilitative), their data confirmed that the severity of physical impairments is reflected in mental rotation abilities (Katschnig et al., 2010). Based on these data it could be concluded that, regardless of the general availability of sensory feedback, the most crucial factor influencing mental rotation is body asymmetry, suggesting that the sensory-motor system tends to put more weight on the available information with a consequent detriment for the representation of the affected body part.

A way to test this possibility takes into account the mechanisms of postural and proprioceptive online recalibration. In healthy subjects, congruent visuo-tactile stimulation promotes self-attribution of a fake hand as explicitly measured by self-reports (“rubber hand illusion”), but does not necessarily affect proprioceptive hand recalibration as implicitly measured by the “proprioceptive drift” procedure (Rohde et al., 2011). Possibly due to such implicit–explicit dissociation, in FHD the illusory self-attribution is preserved but the proprioceptive drift is impaired (Fiorio et al., 2011). However, it is not clear whether the absence of proprioceptive drift in FHD is due to measurement (in)sensitivity or to aberrant sensory-motor plasticity. The possibility to quantitatively measure the behavioral outcomes of the plasticity of sensory-motor representations is provided by mental rotation. Indeed, in healthy subjects the illusory self-attribution due to the rubber hand illusion correlates with the performance in such mental transformations, even in the absence of proprioceptive drift (Ionta et al., 2013). Nevertheless, despite such measurements might provide a less controversial measurement of proprioceptive hand recalibration in FHD, no data are currently available.

## A THEORETICAL MODEL OF HEALTHY AND ABERRANT SENSORY-MOTOR INTEGRATION

**FIGURE 1:**
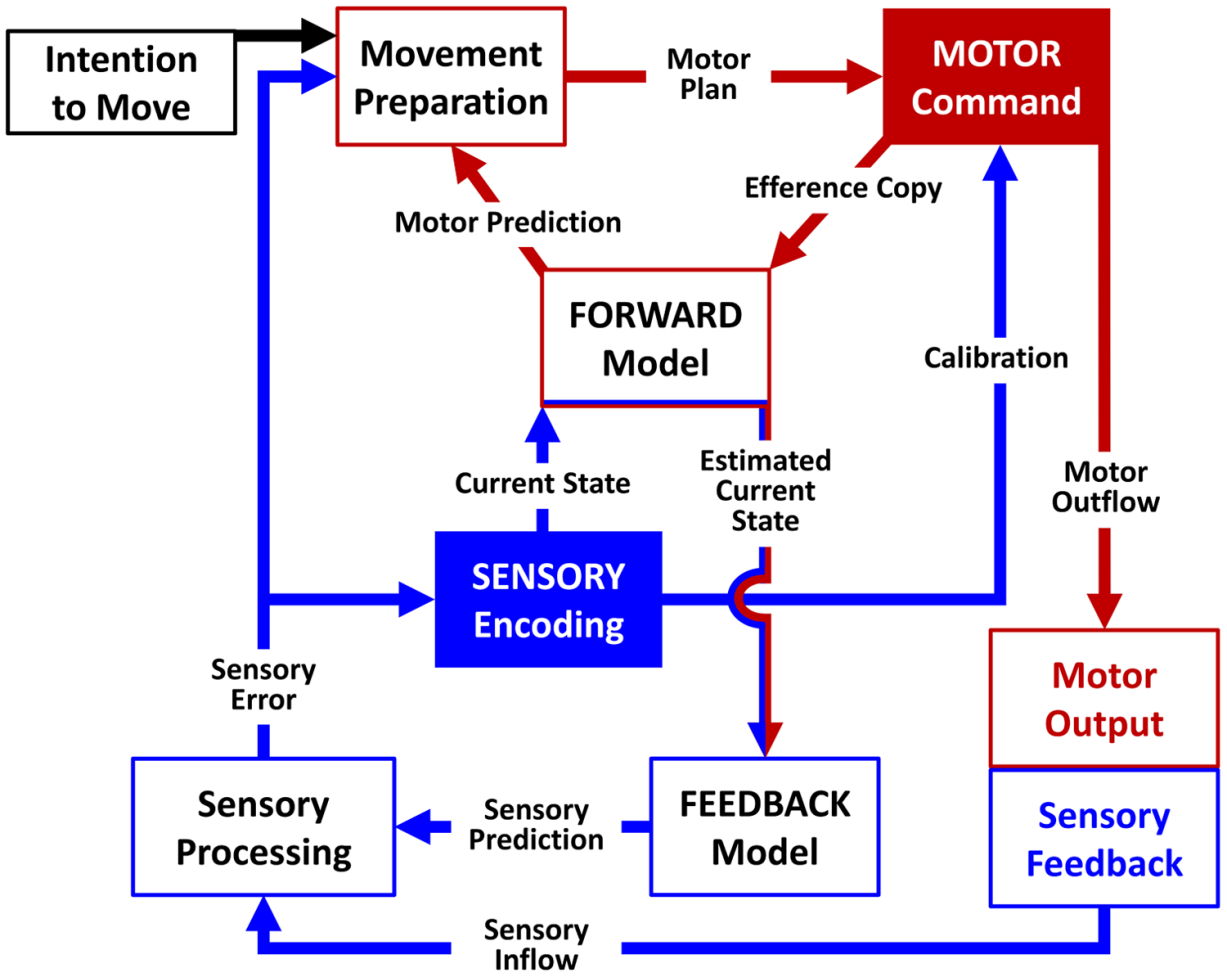
**Schematic representation of the SMILE model**. The signal sent by the motor command node (red arrows) comprises an efference copy processed by the forward model and a motor outflow generating a sensory feedback (blue arrows). Low-level nodes compare the actual sensory feedback to an anticipated sensory prediction generated by a feedback model and transmit information on the resulting sensory error to high-level nodes in order to calibrate the subsequent motor command.

A major challenge in clinical neuroscience is building a model that can explain the causal link between dysfunctional brain networks and particular clinical phenotypes. The available computational models of sensory-motor integration (Wolpert et al., 1995; Sanger and Merzenich, 2000; Shadmehr and Krakauer, 2008) agree on the presence of one or more nodes dedicated to the movement preparation phase. Building on previous computational models, we put forward a biologically based model of sensory-motor integration defined as the Sensory-Motor Integrative Loop for Enacting (SMILE). According to the SMILE model, a proper sensory-motor integration implies the coordination of both high-level and low-level nodes. First, the signals in the high-level preparation nodes are triggered by the intention to move or as a reaction to somatosensory information. These nodes encode the movement preparation phase, and the signals are transmitted to the node where they are converted into motor commands. This node produces the motor outflow and volleys the information to the periphery via the cortico-spinal tract. Simultaneously, the motor command node generates an internal copy of the motor outflow (efference copy) to be further processed by a forward model together with the information on the body state (current state) coming from the high-level sensory encoding node. The role of the forward model is twofold. On the one hand, based on the efference copy it simulates the movement dynamics and predicts the outcome of the motor command (motor prediction). On the other hand, it combines the efference copy and the information regarding the current state in order to enter an estimate of the current state into a feedback model, which in turns creates an anticipation of the sensory consequences of the movement (sensory prediction). When the movement starts, the difference between the anticipated sensory prediction and the actual sensory feedback (sensory inflow) is processed by low-level sensory nodes and is eventually used to correct the current state (sensory error). These sensory low-level nodes would project back to the high-level nodes relative to movement preparation and the sensory encoding. Both the sensory encoding and the movement preparation nodes would in turn project to the motor command node, regulating in this way the balance between the sensory and the motor processes (calibration). Thus the forward and the feedback models are interdependent. The feedback model depends on the estimated current state, which in turn is computed by the forward model taking into account the actual current state. For this reason the relative weight of the estimated and actual sensory effects changes across time. At beginning of the movement the information of the estimated current state is strongly reliable and then sensory-motor integration relies on the forward and feedback models. Towards the end of the movement, the estimated current state is much less reliable and then sensory-motor integration has to rely on the sensory inflow (**Figure 1**).

**FIGURE 2:**
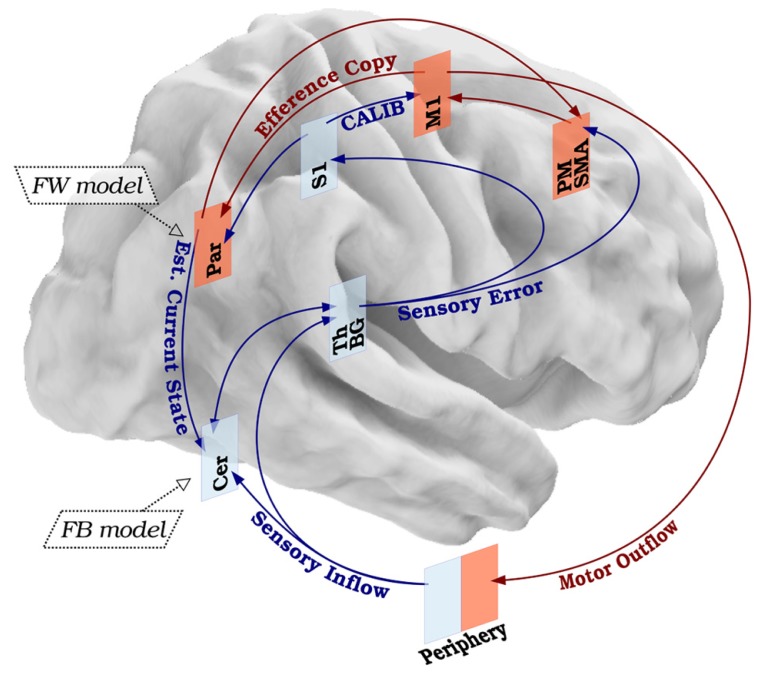
**Biological basis of the SMILE model**. Healthy sensory-motor integration leads to balanced motor command and sensory feedback. The primary motor cortex generates a motor outflow and an efference copy. The motor outflow triggers a sensory inflow, which is processed in low- and high-level modules and contributes in fine-tuning the next motor command. The efference copy is used to anticipate the kinesthetic (forward model) and somatosensory (feedback model) consequences of the movement itself. The difference between the real and expected sensory effects will calibrate the next movement. Red arrows represent motor components. Blue arrows represent sensory components. Legend: S1, primary sensory cortex; M1, primary motor cortex; PM, premotor cortex; SMA, supplementary motor area; Par, parietal cortex; Th, thalamus; BG, basal ganglia; Cer, cerebellum; FW model, forward model; FB model, feedback model; CALIB, calibration; Est. Current State, estimated current state.

Based on the available data, we propose that the SMILE model can represent a way to biologically situate and experimentally test previous computational models of sensory-motor integration in clinical phenomena such as dystonia. At the biological level, according to the SMILE model the movement preparation would be encoded by the premotor and supplementary motor regions (Ionta et al., 2010a). These regions would work as the movement preparation nodes and would exchange information with M1, which would function as the motor command node. When M1 sends the motor command to the periphery, it simultaneously generates an efference copy of the motor outflow which is further processed by the forward model in order to create a motor prediction probably encoded in the parietal cortex (Wolpert et al., 1998). Simultaneously, the forward model contributes to predict the sensory outcome of the movement itself (estimated current state) by entering the information on the actual current state from S1 in the feedback model encoded possibly by the cerebellum (Blakemore et al., 2000). The difference between the anticipated sensory prediction and actual sensory inflow is coded initially by basal ganglia, thalamus, and cerebellum as low-level nodes. Then the signals processed by these low-level nodes would be sent to the primary sensory encoding node (S1), as well as back to the premotor and supplementary motor areas. Working in coordination, these three nodes (S1, premotor, supplementary motor area) would project back to M1, calibrating the subsequent motor output. Thus, through the somatosensory feedback first processed by cerebellum, basal ganglia, and thalamus and then modulated by premotor, supplementary motor area, and S1, the motor execution commands are calibrated in M1, and the loop is closed (**Figure 2**).

**FIGURE 3:**
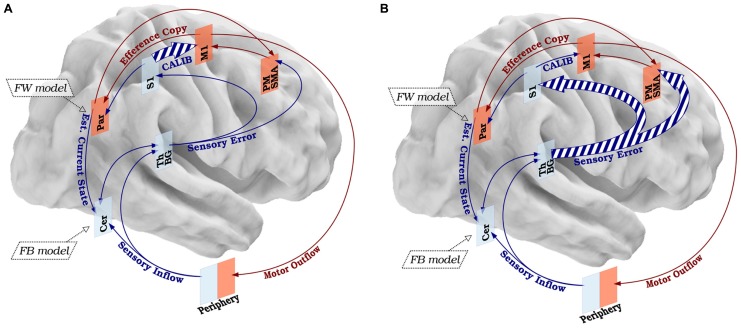
**Disorganization of the SMILE model in FHD. (A)** According to a first hypothetical disorganization, FHD could be the consequence of altered calibration (CALIB) due to abnormal signal sent from S1 and M1, resulting in an aberrant motor command. **(B)** A second hypothesis concerns the possibility that the sensory information is distorted already in the low-level nodes, resulting in an altered signal transmitted from the sensory processing nodes to S1 and the movement preparation nodes (PM-SMA). The dashed lines represent qualitative anomalies in signal processing. The size of the arrows represents the quantitative features of the signal. The legend and color code of **Figure 1** applies to **Figure 2**.

Taking into account the hypothesized mechanisms of FHD and the possible structure described in the SMILE model, we propose that FHD is the manifestation of a breakdown in the sensory-motor loop as the result of a disorganization targeting S1 and due to over-training-related abnormal neuroplasticity, impaired cortico-subcortical dynamics, and local loss of inhibition. Based on evidence that FHD patients exhibit impairments in temporal and spatial discrimination, but not in overt motor behaviors other than the task-specific ones, a first hypothesis is that the breakdown of the sensory-motor integration happens in the high-level nodes, specifically in S1. The breakdown would determine no equivalence between the signal sent from the periphery to S1, and the one sent from S1 to M1 (calibration). When M1 sends the signal to the periphery through the brainstem, the peripheral muscle activation (through the cerebellum, basal ganglia, and thalamus) sends a feedback signal to premotor, supplementary motor and primary sensory regions, which in turn have back projections to equivalent areas in M1. We hypothesize that if the gain of the signals sent through this loop is >1, then M1 keeps increasing its firing until maximal muscle contraction occurs, that is the typical cramp of FHD (**Figure 3A**).

Taking into account the possibility that the deterioration of the sensory information could happen in the low-level nodes and that the whole thalamus-basal ganglia circuit preserves somatotopic organization all along (Vitek, 2002), a second hypothesis is that S1 receives already “disordered” sensory errors from the sub-cortical and cerebellar modules. This would imply that only a fraction of the sensory feedback could be impaired, i.e., the component for the hand, supporting that sub-cortical modules, and thus the feedback from cerebellum–thalamus–basal ganglia complex to S1 (plus the signal from S1 to M1) is impaired and causes problems downstream (**Figure 3B**)

The SMILE model explains (1) task-specific impairments in terms of a breakdown in only some sub-components of the sensory-motor loop, (2) increasing muscle contraction resulting in cramps as a function of the unbalance between sensory input and motor output, and (3) spreading activity to agonist muscles (due to overlapping cortical representations) as a function of extremely repetitive behaviors that would cause cortical disorganization. Taking into consideration this tight association between sensory input and motor output, it is clear how crucial their dissociation is for better understanding the nature of their integration, and therefore the implementation of mental rotation as investigation tool in future experimental protocols.

## CONCLUSION AND FUTURE PERSPECTIVES

Neuroimaging studies based on previous models showed the involvement of both cortical and subcortical regions, suggesting that dystonic deficits affect a broadly distributed network but leaving unsolved the issue of which different nodes of this network are specifically impaired. The inconsistencies in the available results could be due to methodological differences in experimental protocols, required tasks, scanning procedures, or the underestimation of the distorted sensory feedback as a crucial confounding factor that renders the investigation of sensory-motor processes particularly difficult. Conversely, mental rotation of body parts engages anatomically interconnected brain systems implicated in the integration of sensory-motor information and has been implemented with brain imaging for studying the properties of the sensory-motor system in movement disorders such as Parkinson’s disease. However, both neuroimaging and physiological data necessary to identify the pathophysiological characteristics of FHD are still lacking, and mental rotation is a good tool to acquire this information. This important information on brain activity and cortico-spinal communication relative to mental rotation of body parts in FHD represents an unresolved gap that could and should be filled. Finding the influence of FHD in modulating the activity of specific neural circuits, such as hyper-synchronous activity, might help not only to better understand the pathophysiology of FHD but also to develop *ad-hoc* interventions aiming at further regulating those brain circuits.

Rather than a conclusive definition of the pathophysiological mechanisms of the different subtypes of dystonia, the advance brought by the SMILE model is an understanding of the general mechanisms of sensory-motor integration together with the promotion of mental imagery as an experimental approach able to overcome the previous methodological limitations. Such a theoretical–experimental joint approach is essential to obtain the new data required to precisely define the pathophysiology of the different subtypes of dystonia. The lack of this combination is probably one of the reasons why, despite the comprehension of the importance of the sensory components, previous models of movement disorders have pooled together different dystonic symptomatologies (e.g., Patel et al., 2014).

## Conflict of Interest Statement

The authors declare that the research was conducted in the absence of any commercial or financial relationships that could be construed as a potential conflict of interest.

## ABBREVIATIONS

CSP: cortical silent period
FHD: focal hand dystonia
M1: primary motor cortex
S1: primary sensory cortex
SDT: spatial discrimination threshold
SMILE: Sensory-Motor Integrative Loop for Enacting
TDT: temporal discrimination threshold.
